# Investigating muscle protein synthesis using deuterium oxide: The impact of dietary protein interventions across the lifespan

**DOI:** 10.1113/EP092016

**Published:** 2025-04-24

**Authors:** Matthew S. Brook

**Affiliations:** ^1^ Medical Research Council Versus Arthritis Centre for Musculoskeletal Ageing Research University of Nottingham Nottingham UK; ^2^ NIHR Nottingham BRC University of Nottingham Nottingham UK; ^3^ School of Life Sciences University of Nottingham Nottingham UK

**Keywords:** deuterium oxide, protein synthesis, skeletal muscle, stable isotopes

## Abstract

This review highlights recent advancements in our understanding of muscle protein synthesis (MPS) across the lifespan, with a focus on dietary protein strategies to support muscle health. Given that skeletal muscle is crucial for whole‐body metabolism, movement and independence, maintaining muscle mass throughout life is essential. However, the gradual decline in muscle mass and strength with age, known as sarcopenia, represents a significant health concern. Muscle mass is regulated by the balance of MPS and muscle protein breakdown, with dietary protein intake playing a central role in stimulating MPS and maintaining a positive protein balance. Much of our current understanding of protein intake, specifically its quantity, quality and distribution, comes from stable isotope‐labelled amino acid methods. These techniques, however, are limited by time constraints and controlled settings, providing only brief snapshots of MPS dynamics. The use of deuterium oxide (D₂O) has provided new insights, enabling long‐term measures of muscle protein metabolism in free‐living conditions. Measurements of longer‐term MPS using D₂O suggest that older adults might benefit from protein intakes of >1.2 g/kg/day to enhance MPS. Additionally, replacing protein in the diet with higher‐quality sources or enriching lower protein intakes with leucine can further increase MPS. Nevertheless, discrepancies remain regarding optimal protein requirements and the long‐term efficacy of supplementing with enriched suboptimal protein doses. The continued application of D₂O in dietary protein research has the potential to provide further insights into the prolonged effects of various protein strategies on muscle preservation across the lifespan.

## Abstract (highlights)

- **What is the topic of this review?**
  This review discusses our current understanding of the impact of dietary protein intake on muscle protein synthesis and how deuterium oxide has provided new insights into long‐term measures of muscle protein metabolism in free‐living conditions.
- **What advances does it highlight?**
  This review highlights that protein quantity, protein quality and enhancing leucine content can increase long‐term muscle protein synthesis. Furthermore, evidence suggests that protein intake of >1.2 g/kg/day can increase muscle protein synthesis in older adults, and intakes below this benefit from consuming higher quality proteins and supplementing with leucine.

## 1 INTRODUCTION

Not only is skeletal muscle essential for facilitating movement and enabling the completion of daily activities, but it also plays numerous crucial roles in maintaining overall health (Brook, Wilkinson, Phillips, et al., 2016). Accounting for ∼40% of total body mass, skeletal muscle is the largest protein reservoir in the body, from which amino acids (AA) can be mobilized during periods of nutritional deficiency to support vital functions in other organs (Pozefsky et al., 1976; Wolfe, 2006). Additionally, skeletal muscle is a major site for glucose disposal, which plays a crucial role in regulating blood glucose levels and preventing the development of insulin resistance and type 2 diabetes (Defronzo et al., 1985). Given these pivotal roles, maintaining healthy muscle mass throughout life is essential for metabolic health and functional independence (dos Santos et al., 2017). However, many of us will experience some degree of muscle mass loss throughout life that could be related to illness, injury or inactivity (Nunes et al., 2022).

A significant challenge arises with ageing, in which we all experience a gradual decline in muscle mass and strength, a condition known as sarcopenia. Sarcopenia results in muscle mass loss of 0.64%–0.7%/year in women and 0.8%–0.98%/year in men aged 75 years (Mitchell et al., 2012). Overall, sarcopenia is a major contributor to frailty, loss of independence and increased morbidity in older adults (Cruz‐Jentoft et al., 2019). As such, strategies aimed at preserving or restoring muscle mass throughout life and in older adults are of great importance.

A key factor in regulating muscle mass is dietary protein intake, which influences the balance between muscle protein synthesis (MPS) and muscle protein breakdown (MPB) (Atherton & Smith, 2012). Stable isotope tracers have become indispensable in capturing this dynamic balance, overcoming the limitations of traditional static measurements, such as concentration. Unlike static measures that provide only snapshots, stable isotope tracers offer continuous, kinetic insights into metabolic activity (Brook & Wilkinson, 2020). The application of stable isotope‐labelled AA has significantly enhanced our knowledge of muscle metabolism, establishing our understanding of muscle responses to nutrition, activity and ageing (Atherton, Etheridge, Watt, et al., 2010; Brook et al., 2022; Kumar et al., 2009; Wilkinson et al., 2021). However, the resurgence of deuterium oxide (D_2_O) as a stable isotope tracer provides a new perspective for investigating long‐term, free‐living (i.e., incorporating all habitual activity and dietary behaviours) measures of MPS (Brook & Wilkinson, 2020). In this review, I discuss our current understanding of muscle mass regulation and explore the insights gained from applying D_2_O to investigate dietary protein interventions aimed at supporting muscle health across the lifespan, with a focus on age‐related muscle loss.

## 2 STABLE ISOTOPES TO MEASURE DYNAMIC METABOLISM

**FIGURE 1:**
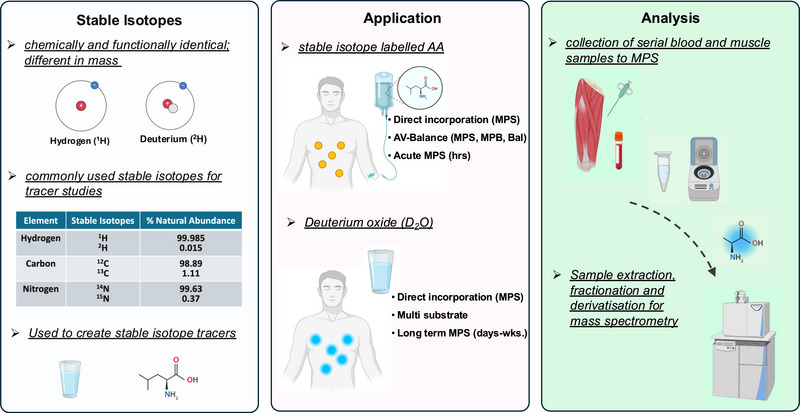
Application of stable isotope tracers to measure muscle protein synthesis (MPS). Stable isotopes are elements with the same number of protons but a different number of neutrons, giving them a difference in mass that is analytically distinguishable while preserving their functional properties. By substituting one or more of the atoms in a compound of interest (e.g., ^12^C or ^1^H) with their heavier, less naturally abundant stable isotopes (^13^C or ^2^H), a stable isotope tracer can be synthesized, such as a stable isotope‐labelled amino acid (AA) or deuterium oxide (D_2_O). This stable isotope tracer enables tracking through biological systems, thereby providing insights into physiological processes. A stable isotope‐labelled AA enables a range of muscle measures, including MPS, muscle protein breakdown (MPB) and arteriovenous (AV) balance. D_2_O can be used for long‐term measures of MPS, alongside the potential to make multi‐substrate measures. Direct incorporation measures of MPS can be made through the collection of serial blood and muscle samples. The samples can undergo processing to measure specific fractions, such as myofibrillar proteins or sarcoplasmic proteins, or can be measured whole as mixed muscle. Subsequently, AA can be isolated from samples and the enrichment determined via mass spectrometry.

Elements are defined by the number of protons in their nuclei, whereas variations in neutron number affect atomic mass without altering chemical behaviour, because neutrons have no charge. Most elements have a predominant isotope, but different numbers of neutrons create isotopes with unique masses (Figure 1). Elements typically possess both stable (non‐radioactive) isotopes and unstable (radioactive) isotopes. The half‐lives of radioisotopes vary widely, impacting their research applications; for instance, ^13^N, with a half‐life of only a few hours, is unsuitable for long‐term studies. In contrast, stable isotopes do not emit radiation and are safe for use in humans, making them ideal for metabolic and physiological studies (Kim et al., 2016; Wilkinson et al., 2021). The mass difference between isotopes renders them analytically distinguishable while preserving their functional properties. By substituting one or more of the atoms in a compound of interest (e.g., ^12^C or ^1^H) with their heavier, less naturally abundant stable isotopes (^13^C or ^2^H), a stable isotope tracer can be synthesized (Figure 1). This stable isotope tracer enables tracking through biological systems, thereby providing insights into physiological processes. For an in‐depth review of the development and application of stable isotope tracers, readers are referred to Wilkinson (2018). Specific stable isotope tracers are available for distinct metabolic pathways; to investigate muscle protein metabolism and MPS typically involves using stable isotopically labelled AA.

### 2.1 Stable isotope‐labelled amino acids

The use of stable isotope‐labelled AA has significantly enhanced our understanding of muscle mass regulation, particularly in response to nutritional intake, physical activity and ageing (Atherton & Smith, 2012). The gold standard for quantifying MPS is via direct incorporation techniques, which typically involve sterile intravenous infusions of labelled AA. Through the collection of serial blood samples and muscle biopsies, the incorporation of a labelled AA into muscle protein over time enables the calculation of a fractional synthesis rate (Wilkinson et al., 2021). The fractional synthesis rate provides insights into different aspects of MPS depending on how the samples are processed. If the entire muscle is analysed, it reflects mixed MPS, whereas fractionation allows for the assessment of myofibrillar (myoMPS) or sarcoplasmic protein synthesis. In addition to direct incorporation techniques, AA tracers can be used for arteriovenous balance methods for the assessment of net muscle protein balance, either in the whole body or across specific limbs and organs (Figure 1; Millward & Smith, 2019). These measures of MPB are typically non‐specific, relying on the dilution of a stable isotope tracer. However, combining these techniques can provide valuable insights into both MPS and MPB. Together, these techniques provide a comprehensive analysis of muscle protein metabolism, capturing dynamic changes in response to various physiological stimuli.

The combination of multi‐labelled stable isotopes with high‐sensitivity mass spectrometry equipment now provides exceptional temporal resolution to investigate acute metabolic responses. These techniques have revealed that skeletal muscle is a dynamic tissue, with a turnover rate of ∼1%–2% per day, modulated by both nutrient intake and physical activity (Atherton & Smith, 2012; Brook, Wilkinson, Phillips, et al., 2016). For instance, in the fasted state MPB exceeds MPS, resulting in a net loss of AA (Biolo et al., 1997; Volpi, 2002). Upon nutrient intake, mixed and myoMPS are transiently stimulated, resulting in the incorporation of dietary AA into muscle protein and replenishing AA lost during fasting (Atherton, Etheridge, Watt, et al., 2010; Bohé et al., 2001). Initial studies established that the protein components of a meal are the primary stimulus for MPS, an effect attributable to the essential amino acids (EAA) within the protein (Volpi et al., 2003). Moreover, the phenomenon of the muscle full effect was identified, whereby myoMPS returns to baseline despite sustained elevations in plasma EAA concentrations (Atherton, Etheridge, Watt, et al., 2010), with maximal stimulation of MPS observed following the ingestion of ∼20 g of protein or 10 g of EAAs (Cuthbertson et al., 2004; Witard et al., 2014). Similar MPS responses have been observed with whole protein sources, such as beef (Symons et al., 2009), in comparison to protein isolates or free AA. However, in certain cases, meals composed of whole foods (particularly with whole plant‐based proteins) can elicit distinct effects on MPS owing to differences in digestion kinetics, AA appearance and nutrient interactions that modulate postprandial AA availability (Pinckaers et al., 2024). Consequently, considerable research has focused on protein type and composition (e.g., animal vs. plant sources), because factors such as the AA profile, digestibility and bioavailability might influence MPS outcomes (Church et al., 2024; Tang et al., 2009). In parallel, postprandial insulin elevation helps to suppress MPB (Greenhaff et al., 2008). These coordinated anabolic responses to feeding help to maintain muscle mass in healthy, weight‐bearing individuals.

Acute AA tracer studies have demonstrated a clear reduction in mixed and myoMPS responses to protein intake in older adults, a phenomenon referred to as anabolic resistance (Cuthbertson et al., 2004; Volpi et al., 2000; Wall et al., 2015). In younger individuals, the ingestion of even moderate amounts of protein (10–20 g) can significantly elevate mixed and myoMPS, contributing to muscle maintenance and growth (Moore et al., 2009; Witard et al., 2014). In older adults, equivalent protein or EAA intake results in a smaller increase in myoMPS, even when plasma AA availability is comparable between age groups (Cuthbertson et al., 2004; Wall et al., 2015). However, anabolic resistance is not always observed (Symons et al., 2008), potentially driven by differences in protein feeding, the population studied and methodological factors, such as variability in biopsy timing and the muscle fraction analysed. Nevertheless, large data sets have demonstrated anabolic resistance in mixed MPS (Wall et al., 2015), with the dose–response relationship between protein intake and myoMPS in older adults displaying a rightward shift, suggesting that larger amounts of protein are required to produce equivalent responses. For instance, in younger adults, ∼20 g [0.24 g/kg body weight (BW), 95% confidence interval 0.18–0.3 g/kg BW] of high‐quality protein is sufficient to stimulate MPS maximally. In contrast, older adults often require 30–40 g (0.4 g/kg BW, 95% confidence interval 0.21–0.59 g/kg BW) to elicit a similar response (Moore et al., 2015). However, many older adults find it challenging to consume large amounts of protein and often consume insufficient protein with each meal (Smeuninx et al., 2020). Furthermore, the quality of the protein source, specifically its leucine content, plays a crucial role in anabolic responses, because leucine is a potent activator of mTORC1 signalling and MPS (Atherton, Smith, Etheridge, et al., 2010; Smith et al., 1992; Wilkinson et al., 2013). The reduced ability of muscle tissue to respond to anabolic stimuli contributes to the steady decline in muscle mass and function observed with advancing age.

Despite the significant advancements made using stable isotope‐labelled AA, some gaps in our understanding remain. For instance, the relationship between acute measures of MPS captured over hours and long‐term muscle adaptations has been questioned. Acute increases in MPS do not always predict long‐term muscle hypertrophy (Mitchell et al., 2015), highlighting the need to characterize the temporal dynamics of MPS better and to use strategies to capture prolonged responses. Moreover, a primary limitation of stable isotope AA tracers is the reliance on costly, sterile intravenous infusions, which confines these studies to controlled laboratory settings and limits their duration, often over 3–6 h (Smith et al., 2011). This restricts the applicability of outcomes to ‘real‐life’ conditions, because controlled environments do not reflect the potential variations in MPS that occur with everyday activities or changes in dietary intake. For instance, muscle anabolic responses are highly influenced by activity levels, with resistance exercise enhancing muscle sensitivity to AA, thereby potentiating MPS (Witard et al., 2014), whereas physical inactivity can induce anabolic resistance (Brook et al., 2022). Additionally, many studies assessing MPS use a single isolated protein source, whereas habitual dietary behaviours involve a complex interplay of meal distribution, timing, nutrient co‐ingestion and the consumption of multiple mixed meals. These factors collectively influence AA availability, with muscle anabolic responses reported over 0–6 h (van Vliet et al., 2019). That being said, AA tracers enable precise measurements of MPS, allowing for the detection of subtle differences in anabolic responses to various protein sources, doses and feeding strategies (Wilkinson et al., 2021). The use of isolated protein sources has been instrumental in establishing fundamental principles of protein metabolism, including digestion and absorption kinetics, AA availability and dose‐dependent effects on MPS (Cuthbertson et al., 2004; Moughan & Wolfe, 2019; Witard et al., 2014). These insights continue to inform research on whole‐food protein sources and complex dietary patterns, reinforcing the crucial role of acute tracer studies in advancing the field of protein nutrition. However, alternative methodologies have been developed that can provide further insight into the repeated impact of a dietary intervention over time to advance our understanding of muscle mass regulation.

### 2.2 Deuterium oxide

**FIGURE 2:**
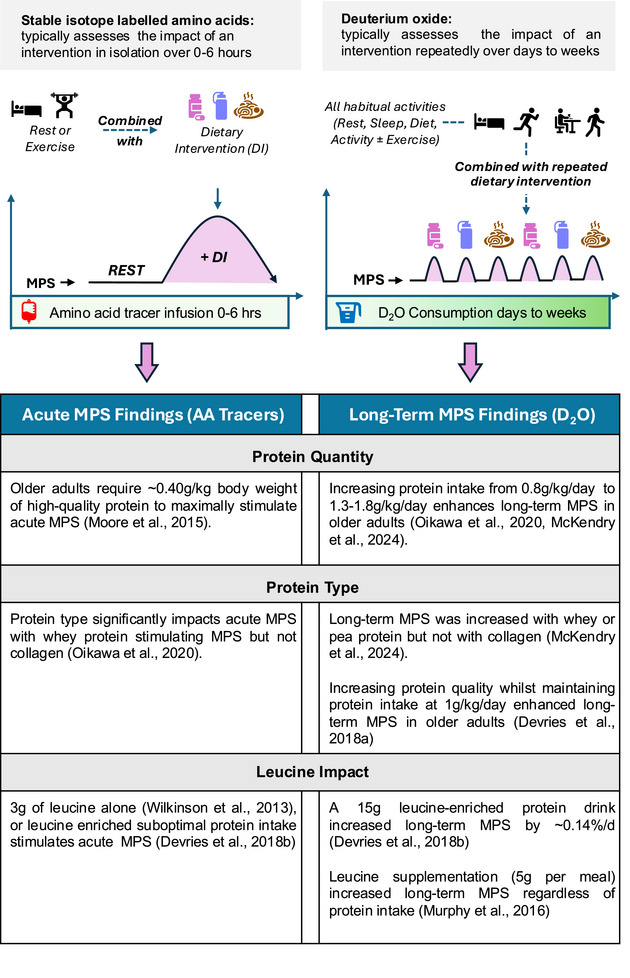
Comparison of acute and long‐term methods for assessing muscle protein synthesis (MPS) responses to dietary interventions. Stable isotope‐labelled amino acid (AA) tracers typically assess short‐term responses to a single intervention (e.g., feeding at rest or combined with exercise), whereas D₂O tracks cumulative MPS over days to weeks, integrating habitual activities and the impact of repeated dietary interventions. The table compares findings from acute and long‐term MPS studies in older adults, highlighting differences in protein quantity and protein type and the effects of leucine supplementation.

Recent advances in analytical instrumentation have led to a resurgence in the use of D_2_O, or ‘heavy water’, as a stable isotope tracer in metabolic research (Brook, Wilkinson, Atherton, et al., 2017; Dufner & Previs, 2003; Holwerda et al., 2024). A significant advantage of D_2_O is its oral administration, which eliminates the need for sterile intravenous infusions, reducing invasiveness for participants in research and clinical settings. Upon ingestion, deuterium rapidly equilibrates throughout the body water (∼2 h in humans; IAEA, 2010); it is incorporated into many biological pathways and labels substrates that can serve as precursors for synthesis (Foletta et al., 2016). For example, AA labelled with deuterium can be used to track protein synthesis, whereas glucose and fatty acids labelled through intermediary reactions can be monitored for carbohydrate and lipid metabolism, respectively (Landau et al., 1996; Previs et al., 2004; Rittenberg & Schoenheimer, 1937). The long half‐life of deuterium in body water, typically ∼9–14 days, depending on individual metabolic rates and water turnover, enables sustained body‐water enrichment (MacDonald et al., 2013; Wilkinson et al., 2014). This prolonged enrichment is crucial for the accurate measurement of MPS over extended periods, allowing cumulative assessments from days to weeks through a single bolus or periodic top‐up dosing strategies (Brook, Wilkinson, Mitchell, et al., 2016; Wilkinson et al., 2014). This capability is particularly valuable for capturing adaptive changes in response to prolonged interventions, such as dietary or exercise regimens (Figure 2).

Various D_2_O dosing strategies have been used, including a single bolus followed by decay (Damas et al., 2016; Wilkinson et al., 2014), a single bolus with regular top‐ups (Brook et al., 2015) or steady‐state maintenance through regular dosing (Holwerda et al., 2018). The choice depends on the study design and analytical sensitivity (Brook, Wilkinson, Atherton, et al., 2017). In short‐term studies, a single bolus is sufficient to measure MPS woing to the long half‐life of D_2_O, which will minimize changes in the precursor pool. In longer studies, weekly top‐ups can reduce participant burden while maintaining body‐water enrichment within a consistent range (Brook et al., 2015). However, maintaining steady‐state enrichment provides more consistent precursor labelling, allowing deuterium incorporation to follow a one‐phase association more closely (Dufner & Previs, 2003). Given that D_2_O captures long‐term MPS, reflecting variations in diet, activity and tracer fluctuations, this might introduce variability over time. Strategies such as a unilateral design, whereby each limb experiences identical conditions, can help to control for this (MacInnis et al., 2017). Further research into the impact of different dosing strategies would offer valuable insights for optimizing protocols across various experimental contexts.

The biochemical mechanisms by which D_2_O quantifies MPS rates are well characterized (Busch et al., 2006; Dufner & Previs, 2003). After ingestion, deuterium equilibrates across intracellular compartments, where it is incorporated into alanine through rapid transamination with pyruvate (Previs et al., 2004). Alanine enrichment reflects 3.7 times the body water deuterium enrichment on average, providing a stable and reliable marker for long‐term MPS (Holwerda et al., 2018; Wilkinson et al., 2014), and is unaffected by changes in diet or activity (Belloto et al., 2007; Dufner et al., 2005). This stability, combined with the capacity of D_2_O to label multiple substrates simultaneously, enables comprehensive metabolic studies, measuring synthesis rates across protein, RNA and DNA (Brook, Wilkinson, Mitchell, et al., 2017; Brook et al., 2023; Busch et al., 2007).

In the following sections, I explore the application of D_2_O in studies on dietary protein interventions, emphasizing any advancements in our understanding of muscle protein metabolism across age.

## 3 EFFECT OF PROTEIN DIETARY INTERVENTIONS ON MUSCLE PROTEIN SYNTHESIS IN YOUNGER ADULTS

In young, healthy adults, muscle mass is typically well maintained (Janssen et al., 2000), and therefore, the application of D_2_O to understand how protein dietary interventions influence the day‐to‐day regulation of muscle mass has been limited in this population. Research has focused primarily on using D_2_O to understand how MPS is influenced by exercise (Brook et al., 2015; Damas et al., 2016; Mallinson et al., 2023) and inactivity (Brook et al., 2022; Kilroe et al., 2020; Shur et al., 2024). However, measuring MPS in young, healthy individuals is crucial for establishing baseline data to understand age‐related disparities. Additionally, long‐term measurements of MPS in young adults could enhance our understanding of how dietary protein impacts muscle regulation over time. Using D_2_O, mixed and myoMPS in young, healthy individuals typically ranges from ∼1.3%/day to 1.7%/day (Brook et al., 2015; Damas et al., 2016; Holwerda et al., 2018; Wilkinson et al., 2014). This measurement incorporates all daily periods of fasting, feeding, resting and activity. These findings align with acute MPS measurements, with rates of 0.05%–0.07%/h equating to 1.2%–1.7%/day (Smith et al., 2011). However, variations across studies are likely, owing to differences in the study population, D_2_O dosing strategies, measurement equipment and precursor used (Smith et al., 2011). Therefore, effect size calculations should be conducted carefully to determine appropriate participant numbers and consider study design factors, such as between‐ or within‐subject designs. Basal rates of MPS should be determined individually for each study, for which a within‐subject design can help to reduce variability and increase statistical power (Houtvast et al., 2024).

The application of D_2_O has been used to investigate different protein types and amounts in younger adults, where the minimal protein requirement is ∼0.8 g/kg/day. Manipulating protein intake over a short period of 3 days had no impact on myoMPS between diets containing high‐protein (1.6 g/kg/day) and low‐protein (0.5 g/kg/day) (Kilroe et al., 2021). However, the prolonged impact of such dietary interventions on MPS remains unknown, with a low‐protein diet likely to have detrimental effects. In addition to protein quantity, protein composition significantly influences acute MPS (Church et al., 2024; Tang et al., 2009). However, consuming a high‐protein diet (1.8 g/kg/day) from either vegan or animal sources demonstrated equal rates of myoMPS over 3 days (Monteyne et al., 2023). Thus, consuming adequate protein levels might offset the effects of inferior protein quality, yet studies of this type have so far been limited by their short duration. With a growing research interest in the identification of sustainable protein sources that meet dietary needs without compromising environmental concerns (Nichele et al., 2022), D_2_O offers opportunities to understand the long‐term effects of protein quantity and quality on MPS.

## 4 EFFECT OF PROTEIN DIETARY INTERVENTIONS ON MUSCLE PROTEIN SYNTHESIS IN OLDER ADULTS

Given the loss of muscle mass associated with ageing (Janssen et al., 2000) and the expanding elderly population (Christensen et al., 2009), there is heightened interest in understanding age‐related muscle atrophy and developing effective strategies to prevent or mitigate this decline. Research has focused increasingly on exploring interventions such as dietary protein, increasing protein quantity, quality and supplementing with AA to enhance MPS.

### 4.1 Increasing protein quantity

With the suggested greater protein requirement to stimulate MPS with age demonstrated in stable isotope infusion studies, D_2_O has been used to investigate higher protein intake for older adults. For instance, older men who consumed 50 g of whey or pea protein (25 g at breakfast and 25 g at lunch) increased myoMPS over 7 days (McKendry et al., 2024). This supplementation was in addition to the current recommended dietary allowance of 0.8 g/kg/day, raising total protein intake to 1.3 g/kg/day and increasing MPS by 0.13%/day (∼9% increase). However, no increase in MPS over 7 days was observed with the supplementation of 50 g of collagen, suggesting that the type of protein plays a crucial role (McKendry et al., 2024). In contrast, older women consuming an additional 250 mL of whole milk, skimmed milk or almond milk on top of an intake of 0.8 g/kg/day did not show increases in myoMPS over 3 days (Stokes et al., 2022). Nevertheless, there was an overall trend towards increased myoMPS with supplementation. This lack of response might be due to sex differences, although the intervention increased protein intake only to ∼1.0 g/kg/day, compared with the 1.3 g/kg/day seen previously (McKendry et al., 2024; Stokes et al., 2022). Similarly, older men on a lower protein diet (0.8 g/kg/day) versus a higher protein diet (1.2 g/kg/day) did not exhibit increases in myoMPS over 3 days (Murphy et al., 2016). As such, it might be that the quantity of protein should exceed 1.2 g/kg/day in older age to increase MPS. Yet, another consideration in the higher protein group is that the distribution of protein intake throughout the day mirrored typical consumption patterns in older adults, providing protein unevenly across meals. Consequently, even the high‐protein group did not meet the 0.4 g/kg body mass suggested to stimulate MPS maximally at most meals (Murphy et al., 2016). Therefore, a more balanced protein distribution throughout the day could have had an effect.

This greater requirement for protein amount and distribution to stimulate MPS is demonstrated by consuming 30 g of whey protein twice a day, once before breakfast and once 1–2 h before sleep. Compared with a maintenance phase of 1 g/kg BW, this increased protein intake to 1.76 g/kg/day, with a more equal distribution throughout the day, and increased rates of myoMPS by 0.063%/day over 6 days (Oikawa et al., 2020). As such, increasing protein intake in older adults at rest might be an effective strategy to stimulate MPS further.

However, without long‐term measures of muscle mass, it is unknown how these elevated rates of MPS reflect increased turnover compared with net protein gain. Furthermore, in many of these studies, protein intake was increased from a baseline of 0.8–1 g/kg BW, and therefore, might only be effective in those consuming lower amounts of protein. Finally, these studies also included an exercise intervention, such as walking or resistance training, that showed much greater increases in MPS. As such, exercise combined with adequate protein is a greater stimulus for muscle growth than protein alone.

### 4.2 Increasing protein quality

In addition to increasing protein quantity, protein quality impacts the stimulation of MPS. Protein quality refers to the ability of a protein to support overall health, determined by factors such as AA composition, digestibility and bioavailability (Cox et al., 2024). High‐quality proteins, such as those found in animal sources, contain all EAA and are easily digestible and bioavailable (Gorissen et al., 2018). For example, increasing protein intake from 1 to 1.87 g/kg/day by consuming 30 g of collagen protein twice a day (once before breakfast and once 1–2 h before sleep) did not enhance myoMPS over 6 days (Oikawa et al., 2020). This finding aligns with previous studies indicating that merely increasing protein quantity without considering quality might not enhance MPS effectively. For instance, consuming 50 g of collagen (25 g at breakfast and 25 g at lunch) did not increase myoMPS in comparison to whey or pea protein in older men (McKendry et al., 2024). Therefore, focusing on both the quantity and quality of protein is essential for optimizing MPS, particularly in older adults who might have increased protein requirements. Acute stable isotope infusion studies demonstrate that high‐quality proteins can stimulate mixed MPS more effectively (Pinckaers et al., 2024), even in smaller quantities (Church et al., 2024), which can help older adults to meet their protein needs without significantly increasing overall food intake. Replacing lower‐quality protein sources with higher‐quality proteins in the diet might provide a strategy to enhance MPS. For example, in a study with older women, replacing 50 g of their daily protein intake with whey protein (25 g twice a day) led to a 3% increase in myoMPS over 6 days (Devries et al., 2018a). The total protein intake was maintained at ∼1 g/kg/day (∼75 g/day), with the substitution resulting in two‐thirds of the daily protein coming from whey.

Another approach to augment MPS involves the use of supplemental leucine to elevate the overall leucine content in smaller protein portions. Acute stable isotope infusion studies identified leucine as a potent anabolic stimulator (Smith et al., 1992), with 3 g of leucine alone demonstrating sufficient capacity to stimulate myoMPS robustly (Wilkinson et al., 2013). The concept of a leucine threshold was initially proposed, suggesting that a specific postprandial plasma concentration of leucine must be reached to stimulate MPS effectively (Tang et al., 2009). However, low doses of leucine‐enriched EAA (1.5–3 g of EAAs containing 0.6–1.2 g of leucine) have also been shown to stimulate myoMPS, indicating that the presence of leucine, rather than the exact amount, is most crucial for anabolism (Wilkinson et al., 2018). Furthermore, leucine metabolites, such as β‐hydroxy‐β‐methylbutyrate (HMB), can stimulate myoMPS without changes in plasma or intramuscular leucine concentrations (Wilkinson et al., 2013). As such, recent evidence has questioned the universality of the leucine threshold (Zaromskyte et al., 2021). Nevertheless, leucine remains a potent stimulator of mTORC1 and can activate MPS directly (Atherton, Smith, Etheridge, et al., 2010; Wilkinson et al., 2013). Additionally, leucine supplementation might confer benefits in specific populations, with a greater impact on older adults (Zaromskyte et al., 2021). Therefore, leucine supplementation might be a viable strategy for older adults, who often find it challenging to consume large amounts of protein.

The use of D_2_O to measure myoMPS in older men showed no difference when consuming a low‐protein diet (0.8 g/kg/day) compared with a high‐protein diet (1.2 g/kg/day) over 3 days (Murphy et al., 2016). However, a significant increase in MPS was observed when each group consumed an additional 5 g of leucine per meal. The peak circulating leucine levels increased approximately fourfold per meal, leading to an absolute increase of ∼0.09%/day (+6% change) in myoMPS. The addition of leucine was equally effective in older men consuming daily protein intakes at the recommended dietary allowance for protein (0.8 g/kg BW/day) and at higher protein intakes (1.2 g/kg BW/day), underscoring the importance of leucine for older men (Murphy et al., 2016). Thus, leucine co‐ingestion with daily meals could effectively enhance integrated MPS in older men, regardless of whether their daily protein intake meets or exceeds the recommended dietary allowance.

Likewise, older women consumed a 15 g milk protein drink containing 4.2 g of total leucine, versus a 15 g protein blend of milk and soy protein isolate containing 1.3 g of total leucine (Devries et al., 2018b). The drinks were consumed twice daily, once in the morning with breakfast and once 1 h before bed. Both groups maintained a dietary protein intake of 1.0 g/kg/day, and myoMPS increased ∼9%/day over 6 days with the leucine‐enriched drink, whereas the control beverage showed no effect. Furthermore, only the leucine‐enriched beverage demonstrated an increase in acute myoMPS, indicating that incorporating leucine into a suboptimal protein dose can enhance both acute and long‐term MPS. Notably, this improvement was observed when the protein drink was integrated into the regular diet, suggesting that this nutritional approach could effectively support muscle anabolism in older adults (Devries et al., 2018b).

In contrast to these findings, older women consumed a lower dose of milk protein (10 g) versus a higher dose of whey protein (25 g), with both drinks containing 3 g of leucine (Devries et al., 2018a). In both groups, the protein drinks were consumed twice daily and were incorporated into a controlled diet that maintained a protein intake of 1 g/kg BW/day. After 6 days, only the whey protein group showed an increase in myoMPS measured using D_2_O. Thus, despite providing a similar amount of leucine, the MPS response was more robust with a higher total protein intake, potentially supporting elevated MPS above basal levels for a longer period after whey protein consumption. Therefore, potentially, leucine‐enriched, suboptimal protein doses might require additional AA to maintain long‐term MPS. For instance, long‐term leucine supplementation alone (2.5 g per meal for 3 months) has shown no significant impact on muscle mass (Verhoeven et al., 2009). However, a leucine‐enriched protein supplement combined with other nutritional factors (20 g of whey protein, 3 g of leucine and 800 IU of vitamin D) has demonstrated benefits on muscle mass (Bauer et al., 2015). As such, although many studies demonstrate that leucine supplementation can enhance MPS, these findings are typically observed over shorter periods (3–6 days); thus, the longer‐term impacts on MPS and muscle mass need to be evaluated further.

Although D_2_O provides additional possibilities for assessing MPS over extended periods, it is important to acknowledge its limitations. D_2_O is used primarily to measure MPS, and whilst it has been applied to measure MPB (Holm et al., 2013), there are alternative methods, such as AV balance (Wilkes et al., 2009) and tracer dilution (d3‐3‐methyl histidine) techniques (Sheffield‐Moore et al., 2014) that might be better suited. Furthermore, the use of substrate‐specific tracers enables the determination of the overall balance of whole‐body protein metabolism, which includes synthesis, breakdown, oxidation and net balance. Moreover, using D_2_O in isolation does not clarify whether increases in synthesis result in net protein accretion or simply reflect elevated protein turnover associated with muscle remodelling. Combining D_2_O with accurate muscle size measurements and tracer dilution techniques might, therefore, enhance our interpretation of net protein gain. Consideration must also be given to the duration of labelling and the chosen D_2_O dosing strategy, which should align with the expected protein turnover rate and the sensitivity of the analytical methods (Brook, Wilkinson, Atherton, et al., 2017). Short labelling periods might result in insufficient signal, whereas prolonged exposure can lead to plateauing, limiting accurate kinetic analysis. It has been suggested that an optimal replacement range of 25%–50% of newly synthesized proteins enables the detection of meaningful changes within a 10%–75% range (Holmes et al., 2015). In addition, the measurement period is crucial for capturing intervention responses. For instance, acute studies using stable isotope AA show that feeding induces significant MPS changes within 6 h, while resistance exercise effects last 48 h or more. However, small differences in dietary response detected with AA tracers might reflect only a fraction of daily fluctuations. Given that D_2_O integrates overall behaviour (Figure 2), its sensitivity to detect minor variations might be limited in long‐term measures. Therefore, precise sampling timing and the use of multiple tracer techniques are essential to capture the metabolic response to a stimulus fully. These considerations underscore that although D_2_O is a valuable tool for longitudinal MPS studies, future research incorporating multiple assessments of protein balance could provide a more holistic understanding of protein metabolism.

## 5 CONCLUSION

In conclusion, advances in stable isotope tracers, particularly D_2_O, have significantly enhanced our ability to investigate MPS across different age groups and dietary conditions in free‐living settings. Studies indicate that both the quantity and the quality of dietary protein are crucial for increasing MPS effectively in older adults. Evidence suggests that protein intake of >1.2 g/kg/day is generally effective for stimulating MPS, although intakes below this threshold can still benefit from a higher proportion of high‐quality, leucine‐rich proteins. (Figure 2). However, discrepancies remain regarding optimal protein requirements and how increased MPS translates to long‐term muscle health outcomes. Although leucine‐enriched, lower protein doses (e.g., 10–15 g protein) can stimulate MPS, evidence suggests that greater AA availability might be required to sustain muscle anabolism over time. Yet, as older adults also face challenges in achieving sufficient protein intake consistently, using these strategies to optimize protein distribution, focusing on high‐quality sources and co‐ingesting supplements might offer additional long‐term benefits for muscle mass and function.

Although significant progress has been made in understanding MPS, future research should strengthen MPS links to functional outcomes and strategies for sustaining muscle health. Although short‐term MPS responses indicate anabolic potential, they do not always predict hypertrophy or strength gains. To address this, studies should implement longitudinal designs that track MPS throughout interventions, alongside measures of muscle mass (dual‐energy X‐ray absorptiometry or MRI) and functional outcomes (strength, mobility or grip strength). This approach might help to identify optimal windows for capturing MPS and improve our understanding of its relationship to muscle adaptation. Additionally, individual variability in MPS responsiveness, driven by metabolic and lifestyle factors, should be explored to refine protein intake strategies and investigate the synergy between exercise and protein metabolism. These efforts will be crucial for translating research into practical dietary and exercise interventions that support muscle maintenance and healthy ageing. The continued application of stable isotope tracers, such as D_2_O, will be essential for exploring the long‐term impacts of dietary protein interventions on MPS, helping to refine dietary guidelines aimed at preserving muscle health and functional independence in ageing populations.

## AUTHOR CONTRIBUTIONS

Sole author.

## CONFLICT OF INTEREST

None declared.
